# Effect of Wireless Channels on Detection and Classification of Asthma Attacks in Wireless Remote Health Monitoring Systems

**DOI:** 10.1155/2014/816369

**Published:** 2014-02-10

**Authors:** Orobah Al-Momani, Khaled M. Gharaibeh

**Affiliations:** Yarmouk University, Irbid 21163, Jordan

## Abstract

This paper aims to study the performance of support vector machine (SVM) classification in detecting asthma attacks in a wireless remote monitoring scenario. The effect of wireless channels on decision making of the SVM classifier is studied in order to determine the channel conditions under which transmission is not recommended from a clinical point of view. The simulation results show that the performance of the SVM classification algorithm in detecting asthma attacks is highly influenced by the mobility of the user where Doppler effects are manifested. The results also show that SVM classifiers outperform other methods used for classification of cough signals such as the hidden markov model (HMM) based classifier specially when wireless channel impairments are considered.

## 1. Introduction 

The application of wireless telemedicine in monitoring patients is seen as a useful and potentially powerful tool to help patients seek medical treatment [1, 2]. In remote monitoring systems of asthmatic patients, cough signals (as well as other body vital signs) are collected by sensors attached to the patient and sent to a PC located in a hospital using wireless technology [3–7]. A classification algorithm is then applied to the received signals in order to decide the state of the patient. Doctor intervention can then be done by calling the patient to take precautions, take a certain medication, or to go to a health care centre for proper treatment.

One of the main purposes of such systems is to help patients take precautions in the case of asthma attacks [4]. An asthma attack is a sudden worsening of asthma symptoms caused by an exposure to allergens or irritants such as inhaling dry and cold air and certain allergens such as pets, pollen, dust, and smoke. The symptoms of asthma attack may vary in severity and duration from person to person. The main symptom that indicates an asthma attack is coughing which has different frequency and strength from regular coughing [4]. Other signs of Asthma attack include headache, blue colour in skin, difficulty in talking, and difficulty in breathing. When these signs of asthma attack are noticed, a patient should immediately seek medical treatment in order to prevent severe asthma attacks which may cause death [5].

With wireless transmission, channel impairments such as amplitude variations, time dispersion, and Doppler effects result in degradation of the receiver ability to recover the transmitted signal. This means that the classification accuracy of medical signals transmitted over wireless channels will be deteriorated as a result of errors in the received data. Unlike voice data, medical signals are more influenced by channel impairments because these signals have low bandwidths and are very sensitive to channel impairments [8]. Therefore, it is important to relate the classification accuracy and hence diagnosability of body signs to channel parameters in order to identify the channel conditions under which transmission is not recommended from a clinical point of view.

This problem has been investigated in the literature where the objective was to determine the transmission conditions where diagnosability of received signals is possible [8–11]. For example, it was shown in [8] that successful transmission of ECG is highly influenced by the mobility of the transmitter where it was shown that the receiver BER exceeds the acceptable limit required for correct classification and diagnosability of ECG signals at speeds above 50 Km/Hr. In [9], a wireless telemedicine system for transmission of photoplethysmography (PPG) signals was analyzed where it was shown that successful transmission of medical data over the tested channels requires a receiver bit error rate of less than 10^−7^.

In this paper, support vector machines (SVM's) are used for classification of cough signals transmitted through a wireless channel in order to make decision about the occurrence of asthma attacks of asthmatic patients who are free to move. SVM classifiers have extensively been used in classification of speech-like signals and were shown to provide higher classification capability for classification of cough than other techniques [12, 13].

The performance of the SVM classification algorithm in detection of asthma attacks is evaluated by computing the probability of correct classification at different channel conditions (channel models) and is compared to the performance of hidden markov model (HMM) classifier [14]. The classification accuracy is related to the channel SNR in order to determine the channel conditions under which the classifier produces acceptable results for detection of asthma attacks.

## 2. Wireless Remote Health Monitoring System for Asthma Patients

**Figure 1:**
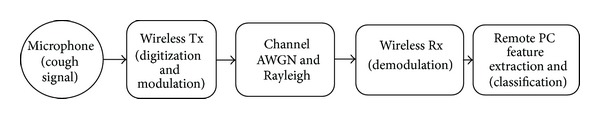
Block diagram of a wireless monitoring system for asthma attack detection.

Figure 1 shows a system model for a wireless remote health monitoring system which can be used for detection of asthma attacks of patients who are free to move. In this system, cough data are captured by a microphone and then transmitted through a wireless channel. At the receiver side, the received signal is demodulated and then entered into a feature extraction block. Extracted features of the received signal are used by the classification algorithm which makes a decision about the existence of an asthma attack.

One of the main challenges in the design of wireless remote health monitoring system is the reliability of the communication channel [13]. Communication channels introduce impairments to the transmitted signal which result in the inability of the receiver to recover the original signal correctly. Hence, errors introduced by the wireless channel impact signal classification and result in wrong interpretation of medical data.

There are two main types of signal degradation introduced by wireless channels: the first is attenuation and random variation of signal amplitude, and the second is distortion of the signal spectrum. Signal attenuation results from the degradation of the signal power level over distance while random variation of signal amplitude results from channel noise and multipath fading effects. Noise effects are modelled by additive white Gaussian noise (AWGN) with a power spectral density that depends on the channel signal-to-noise ratio (SNR).

With AWGN channel model, white Gaussian noise is added to the transmitted signal based on a specified SNR; therefore, the received signal can be expressed as

(1) $$\begin{array}{c} r\left(t\right)=s\left(t\right)+n\left(t\right), \end{array}$$

where  *s*(*t*)  is transmitted signal and  *n*(*t*)  is a noise signal. The noise signal is assumed to be statistically independent of  *s*(*t*), stationary Gaussian noise process with zero mean and two-sided PSD of *N*_0_/2 Watts/Hz. The AWGN model is particularly simple to use in the detection of signals and in the design of optimum receiver in most communication systems.

Random variations of signal amplitude due to multipath fading effects are usually modelled by the Rayleigh channel model [14, 15]. In a Rayleigh channel model, signals from different paths having different phases and similar signal strengths are received to produce a Rayleigh distributed signal amplitude. The received signal from a Rayleigh fading channel is modelled as [13, 15]

(2) $$\begin{array}{c} r\left(t\right)=h\left(t\right)s\left(t\right)+n\left(t\right), \end{array}$$

where  *h*(*t*)  is the fading amplitude which has a Rayleigh probability density function (PDF) given by

(3) $$\begin{array}{c} f_{h}\left(h\right)=\frac{h}{\sigma^{2}}e^{-h^{2}/2\sigma^{2}},\;h\geq 0, \end{array}$$

where  *σ* ^2^  is the average received power and *h* is the signal magnitude.

Distortion of signal spectrum is usually attributed to two main phenomena: the first is multipath delay and the second is the Doppler effects. Multipath delay results from multipath phenomena in wireless channels where the received signal consists of multiple reflected signal components which have different delays. Delay distortion results in intersymbol interference (ISI) where successive symbols interfere and have significant effects on transmitted signals when their bandwidth is larger than the coherence bandwidth of the fading channel (frequency selective channels) [16]. The Doppler spread is a measure a spectral broadening caused by time varying nature of the channel. Doppler spread is usually related to the mobility of either the receiver or the transmitter and is defined as the frequency range in which the frequency of the received signal changes due to Doppler effects. Doppler shift leads to signal distortion which causes errors in the received signal. The Doppler power spectrum for a narrowband fast fading channel is modelled as [16]

(4) $$\begin{array}{c} S\left(f\right)=\frac{1}{\pi f_{D}\sqrt{1-{\left(f/f_{D}\right)}^{2}}},\;\left|f\right|\leq f_{D}, \end{array}$$

where *f*_*D*_ is the maximum Doppler shift introduced by the channel which is linearly related to the speed of either the transmitter or the receiver. The Doppler spectrum specifies the frequency spectrum of the Rayleigh fading signal and hence its autocorrelation function.

Since medical signals usually have smaller bandwidths than the channel coherence bandwidth, the delay spread can be neglected. On the other hand, small variations in signal spectrum due to Doppler spread can cause significant distortion to the transmitted signal when the signal bandwidth is small. Therefore, in the simulations that will follow, delay spread will be neglected and the performance of the classification system will be tested under different values of the Doppler spread.

## 3. Support Vector Machine (SVM) Classification 

SVM classifiers have extensively been used in classification of speech-like signals and were shown to provide higher classification capability for classification of cough than other techniques [12].

SVM is a classification algorithm that performs a classification task by constructing a hyperplane in a multidimensional space that separates data into two different categories. An SVM classifier consists of *L* training points, where each input **x**_*i*_ has *D* dimensions and belongs to one of two classes *H*_1_ and *H*_2_ which correspond to *y*_*i*_ = −1 or +1, where *y*_*i*_ denotes the classifier output. Training data can be represented in the form {**x**_*i*_, *y*_*i*_} where *i* = 1,2,…, *L* and  **x** ∈ *R* ^*D*^  [17]. If the data is linearly separable, then classification of a data point  **x**  is done by maximizing the margin separating the two hyperplanes, which results in a decision rule of the form [18]:

(5) $$\begin{array}{c} \text{Decide}\;\text{Class}\;1\;\text{if}\;\text{ sign }\left(\sum_{i=1}^{L}\alpha_{i}x_{i}y_{i}\cdot x+b\;\right)=1,\\ \text{Decide}\;\text{Class}\;2\;\text{if}\;\mathrm{sign}\left(\sum_{i=1}^{L}\alpha_{i}x_{i}y_{i}\cdot x+b\;\right)=-1, \end{array}$$

where sign(·) is the sign function and *α*_*i*_ are Lagrange multipliers and are referred to as the support vectors (SV) [18].

When data is not linearly inseparable, a kernel function is used to map data to higher dimension such that the resulting data is linearly separable [18]. The original formulation of the SVM classifier remains the same except that every dot product in (5) is replaced by a nonlinear kernel function. Therefore, with using a kernel function, the decision rule in (5) is reformulated as [18]

(6) $$\begin{array}{c} \text{Decide}\;\text{Class}\;1\;\text{if}\;\mathrm{sign}\left(\sum_{i=1}^{L}\alpha_{i}y_{i}K\left(x_{i},x\right)+b\;\right)=1,\\ \text{Decide}\;\text{Class}\;2\;\text{if}\;\mathrm{sign}\left(\sum_{i=1}^{L}\alpha_{i}y_{i}K\left(x_{i},x\right)+b\;\right)=-1, \end{array}$$

where *K*(**x**_*i*_, **x**) is the kernel function which has different forms depending on the application. The most common kernel functions are the polynomial, the radial basis function (RBF), and the sigmoid kernel functions [19].

The parameters of the SVM classification algorithm (including the parameters of the kernel function) can be found using cross-validation of the available training data where the parameters that give the best classification accuracy are selected [17].

## 4. Asthma Attack Detection Using SVM Classification

Figure 3 shows the SVM classification model used in this paper for detection of asthma attacks. The classification process involves extraction of features, generation of a classifier data base from a training set, and decision making for a given test signal.

### 4.1. Feature Extraction

**Figure 2:**
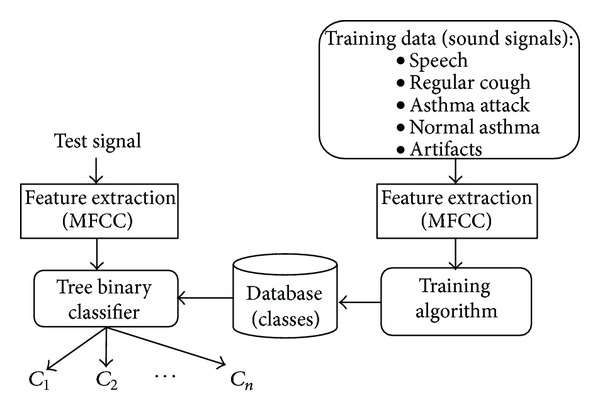
Classifier model.

In the classification model in Figure 2, features of cough signals are extracted and then used by the classification algorithm in order to reduce the dimensionality of the classification problem. Feature extraction can be done using different methods, such as Fourier transform and Wavelet transform-based methods [21].

One of the common Fourier transform-based methods is the Mel Frequency Cepstral Coefficient (MFCC) technique which has been used extensively in feature extraction of speech-like signals [22]. MFCC is a type of parametric representation of speech-like signals and has been extensively used in speech recognition because of its ability to capture relevant information of human speech [20]. MFCC feature extraction process is known for its robustness against time varying nature of speech signals which is a desired feature when processing cough data from asthmatic patients.

**Figure 3:**
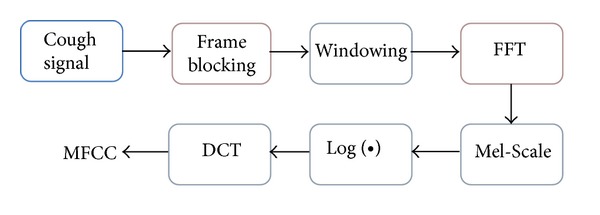
Block diagram of feature extraction process using MFCC [20].

MFCCs are the coefficients that represent the short-term power spectrum of a signal obtained from linear cosine transform of the log power spectrum on a nonlinear “Mel” scale of frequency [22]. The process of calculating MFCCs is illustrated in Figure 3. Digitized cough data is first processed by the framing block which performs segmentation of the cough signal samples into *N* frames. Windowing is applied to the resulting frames before being spectrally analyzed using the fast Fourier transform (FFT). The power spectrum is mapped onto the Mel scale using the approximation [20]:

(7) $$\begin{array}{c} f_{m}=2595\;\text{lo}{\text{g}}_{10}\left(1+\frac{f}{700}\right), \end{array}$$

where  *f*  (Hz) is the normal frequency and *f*_*m*_ is the Mel frequency. MFCCs are calculated from the discrete cosine transform (DCT) of the log of the signal spectrum which is equivalent to passing the Mel scaled log spectrum through a bank of triangular shaped bandpass filters, distributed along a Mel scaled frequency band of interest [14].

### 4.2. MultiClass Classification of Cough Signals

The initial form of SVM classifier is binary as clear from (1) and (2) where the output of the learned function is either positive or negative. Asthma attack detection requires classification of cough signals into five classes: normal cough, cough related to asthma attack, cough related to a regular asthma episode, speech, and artefacts (sounds from patient daily activity including drinking, laughing, clearing through, and crying). To generate multiclass SVMs from binary SVMs, a number of methods have been proposed in the literature [23–25]. One of the most popular methods is the binary tree support vector machine (BTSVM) which combines SVM and binary trees. BTSVM decomposes an *N*-class problem into *N*–1 subproblem, each separating a pair of classes with SVM classifier. BTSVM has a number of characteristics such as lower number of binary classifiers and faster decision speed [23].

Figure 5 shows a tree-based classification process of cough signals. The classifier uses four stages of classification for classifying a sound signal into the five classes mentioned above. The training set for the tree classifier is generated by dividing the database into two disjoint groups at each classification stage.

### 4.3. Kernel Function Selection

Given the diversity of the sound signals incorporated in the training set of the classifier, training data constitutes a linearly inseparable database. Therefore, an RBF kernel function is used as in (2). The RBF kernel has less hyperparameters and less numerical complexity than other kernel functions [17]. The RBF kernel function is described by [18]

(8) $$\begin{array}{c} K\left(x_{i},x_{j}\right)=e^{\left(-{\left|x_{i}-x_{j}\right|}^{2}/{2\sigma }^{2}\right)}. \end{array}$$

The parameter  *σ*  determines the area of influence of the support vector (SV) over the data space. Large value of  *σ*  will allow a SV to have a strong influence over a large area and reduces the SV, but reducing the SV results in increased error [17].

## 5. Simulation and Verification

In the model shown in Figure 6, test signals are transmitted through the wireless channel after performing analogue to digital (A/D) conversion and digital modulation. At the receiver, the signal is converted back to the analogue domain after digital demodulation and then applied to the classifier to make a decision about the existence of asthma attacks.

The performance of the classification system under wireless channel impairments is quantified by computing the probability of correct classification (*P*_*c*_) (the classification rate). The probability of correct classification (*P*_*c*_) is defined as the ratio of the number of correctly classified samples and the total number of the samples [19]:

(9) $$\begin{array}{c} P_{c}\left(\%\right)=\frac{\text{Numbr}\;\text{of}\;\text{correctly}\;\text{classified}\;\text{samples}}{\text{Total}\;\text{number}\;\text{of}\;\text{samples}}\times 100. \end{array}$$

### 5.1. Data Collection

The classification system was tested using recordings of cough signals from real asthmatic patients. The recordings were obtained from 18 patients at the King Abdullah University Hospital (KAUH) in Jordan in order to develop the training set of the classifier (Data was obtained after getting the consent of patients through the hospital procedure and policies). The causes of cough in these patients were as follows: 10 patients had cough caused by asthma and 8 patients had cough caused by an asthma attack. All recording were sampled at frequency 11025 Hz and were divided into samples where each patient produces at least 5 samples or 6 samples. On the other hand, 144 samples from normal subjects including normal cough, speech, and artefacts (drink, laughter, clearing through, and crying) were obtained from http://www.freesound.org/ [26].  Table 3 shows the five types of sound signals used for training the classifiers. The recordings were digitized at a frequency of 11025 samples/second and encoded at 16 bits per sample.

**Figure 4:**
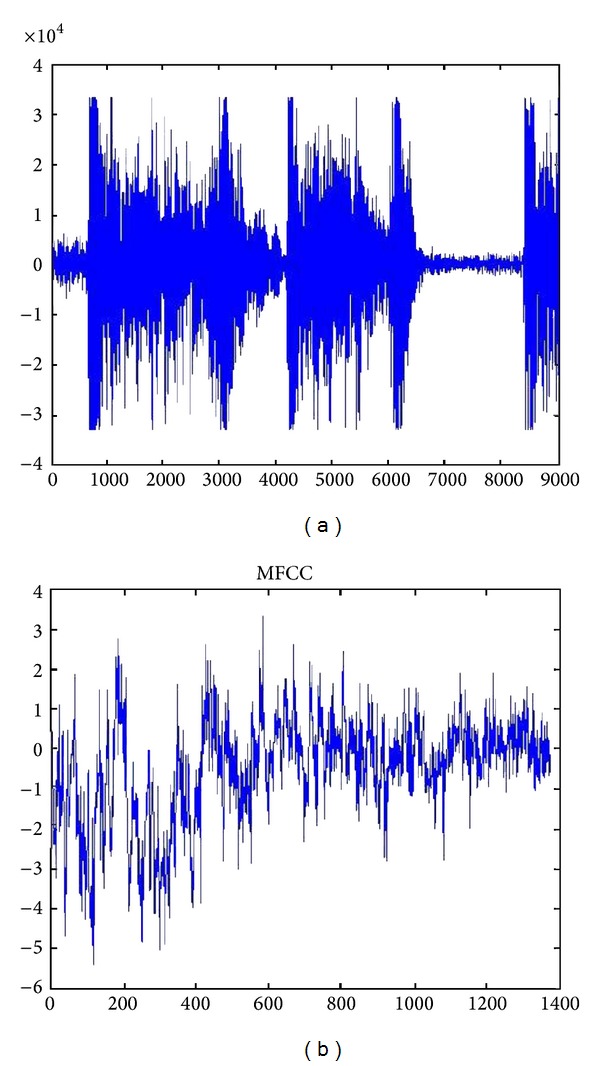
(a) A cough signal of an asthmatic patient and (b) its MFCC.

**Figure 5:**
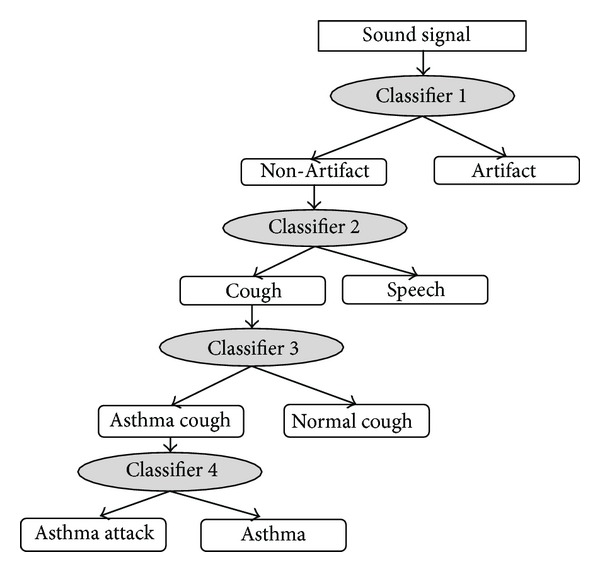
Tree SVM classifier for detection of asthma attacks.

Table 1 shows the parameters used for feature extraction of cough signals using MFCC as discussed in the previous section. Figures 4(a) and 4(b) show a cough signal and its MFCC features.

### 5.2. Wireless Channel Models

Two channel models are considered: an AWGN channel model and a Rayleigh fading channel model. With AWGN channel, noise is simply added to the transmitted signal using the Matlab function “awgn” function where SNR can be specified. A Rayleigh fading channel model is generated using the sum-of-sinusoids method in which complex Gaussian noise with a power spectral density (PSD) that is equal to the Doppler power spectrum in (8) is approximated by a finite sum of weighted sinusoids as [27]:

(10) h(t)=2M∑n=1Mcos⁡⁡(ωdtcos⁡αn+φn) +j2M∑n=1Mcos⁡⁡(ωdtsinαn+φn),

where *α*_*n*_ = (2*πn* − *π* − *θ*_*n*_)/4*M*,  *n* = 1,2,…, *M*, *ω*_*d*_ is the maximum angular Doppler frequency, *φ*_*n*_ and *θ*_*n*_ are independent random variables uniformly distributed on [−*π*, *π*] for all *n*. With this formulation, the fading amplitude (|*h*(*t*)|) approximates a Rayleigh random variable with a PDF as in (7). The parameters of the channel model in (10) used in the simulations which will follow are shown in Table 2.

**Figure 6:**
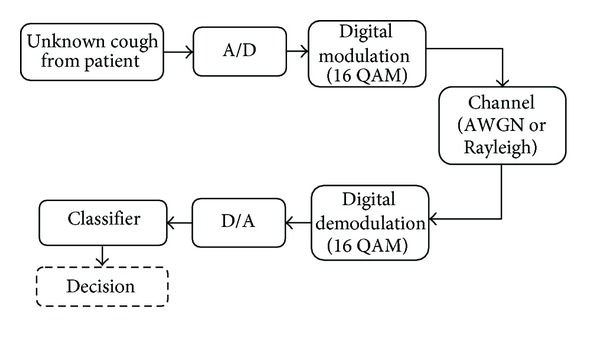
Block diagram of simulation model.

In the simulation model shown in Figure 6, test signals are transmitted through both channel models at different SNRs and Doppler shifts and the number of correctly classified samples is counted. The minimum SNR required for an acceptable *P*_*c*_ is determined for both channel models. Furthermore, in the case of Rayleigh fading channel, the maximum Doppler frequency that results in acceptable classification rate is determined at a given SNR.

### 5.3. Simulation Results

**Figure 7:**
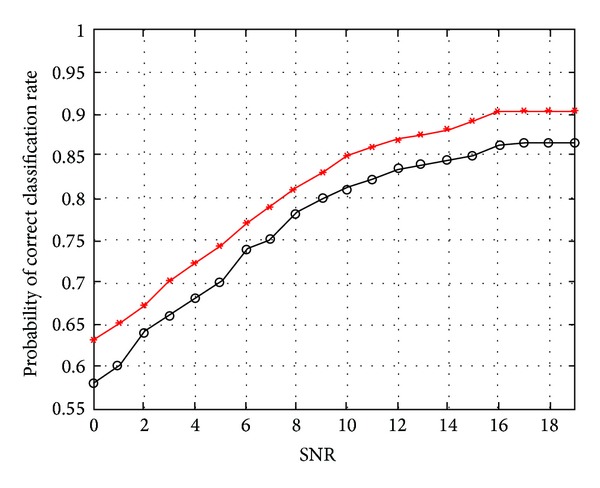
Probability of correct classification in AWGN channel versus SNR; *SVM and °HMM.

Using the AWGN channel model, the average classification rate of the SVM classifier was computed at different SNRs and compared to the classification rate obtained from using the hidden markov models approach in [14].  Figure 7 shows *P*_*c*_ versus SNR for the SVM classifier and compared to that of an HMM classifier.

**Figure 8:**
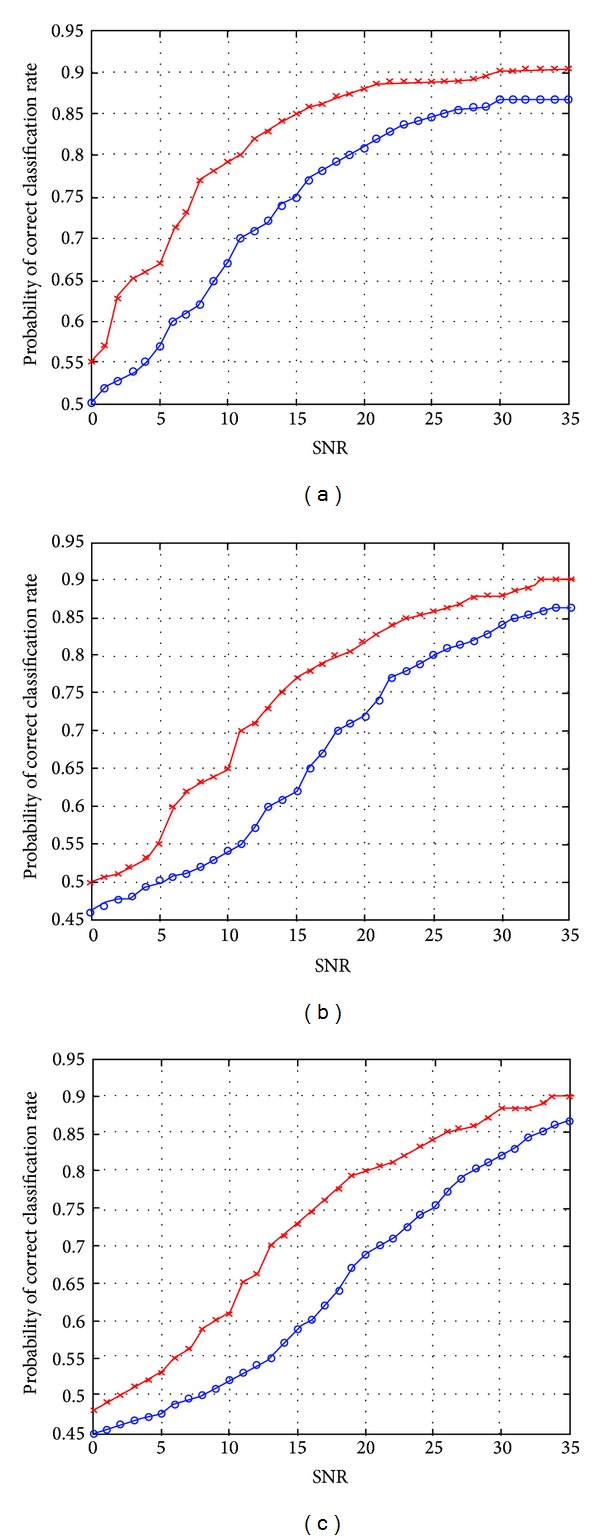
Probability of correct classification in Rayleigh fading channel versus SNR (a) *f*_*D*_ = 0 Hz, (b) *f*_*D*_ = 10 Hz, and (c) *f*_*D*_ = 100 Hz; *SVM and °HMM.

Under a Rayleigh channel model, *P*_*c*_ is calculated at different SNRs and different values of the Doppler shift of the channel. Figures 8(a)–8(c) show *P*_*c*_ versus SNR at Doppler shifts of 0, 10, and 100 Hz of the SVM classifier and compared to the HMM-based classifier.

### 5.4. Discussion

In the case of AWGN channel, the simulation results show that a maximum *P*_*c*_ of 90% is obtained for the SVM classifier at SNR = 16 dB and 86% for the HMM-based classifier at SNR = 17 dB. The results show that the SVM classifier outperforms the HMM classifier at all SNRs.

In Rayleigh fading channel, the simulation results show that the SVM classifier outperforms the HMM classifier at all Doppler frequency shifts. From Figures 8(a)–8(c), the maximum *P*_*c*_ of both classifiers is obtained at SNRs above 33 dB which is about 14 dB higher than the case of using an AWGN channel. This means that SNR needs to be increased in order to overcome the loss in *P*_*c*_ due to the mobility of the transmitter. In general, the simulation results show that the SVM classifier outperforms the HMM classifier when channel impairments are considered. The maximum difference in *P*_*c*_ between the two classifiers is about 17% at *f*_*D*_ = 100 Hz and SNR = 15 dB.

## 6. Conclusion

In this paper, classification of cough signals in a wireless remote health monitoring scenario using SVM classification algorithm has been analyzed. The reliability of the classification system has been tested under different wireless channel models (AWGN and Rayleigh fading) where it has been shown that channel impairments have a significant effect on the accuracy of the classification system. The simulation results show that SVM classification is capable of detecting asthma attacks from cough signals transmitted through a wireless communication channel provided that the channel SNR is increased to overcome the channel impairments caused by noise, amplitude variations due to fading, and the mobility of the wireless transmitter at the patient's side. The simulation results also show that SVM classifier provides better classification accuracy than the HMM-based classifier under the same channel conditions. The results presented in this paper can be used in the design of remote health monitoring systems for asthmatic patients where system parameters can be designed considering the minimum requirements for channel SNR required for achieving the desired classification accuracy.

## Figures and Tables

**Table 1:** MFCC parameters used in feature extraction of cough signals.

Parameter	Value
Number of MFCC coefficient	20
Window	Hamming: *w*(*n*) = 0.54 − 0.46cos⁡(2*n*/(*N* − 1))
Window length (*N*)	256
Frame size	25 ms
FFT size	256
Feature vector dimension	69

**Table 2:** Fading channel parameters.

Modulation order *M *	8
Fading sequence length (*N*)	180019
*φ*_*n*_, *θ*_*n*_	Uniformly distributed random variables *φ*_*n*_ = *θ*_*n*_ = 2 ∗ pi ∗ rand (1, *N*) − pi;
Doppler shift	0, 10, 100 Hz
SNR	0–35 dB

**Table 3:** Sound signals used in generating the training set of the classifier.

Class	Number of samples
Artefacts	48
Speech	48
Normal cough	48
Asthma cough	48
Asthma attack	48
Total	**240**
